# Do NSAIDs Take Us Away From Treatment Goals in Axial Spondyloarthritis: A Story About Dysbiosis or Just a Matter of Bias?

**DOI:** 10.3389/fmed.2021.817884

**Published:** 2021-12-24

**Authors:** Rubén Queiro-Silva, Andrea García-Valle, Sara Alonso-Castro, Mercedes Alperi-López

**Affiliations:** ^1^Rheumatology Division and Instituto de Investigación Sanitaria del Principado de Asturias Translational Immunology Section, Hospital Universitario Central de Asturias, Oviedo, Spain; ^2^Oviedo University School of Medicine, Oviedo, Spain; ^3^Rheumatology Division, Complejo Asistencial Universitario de Palencia, Palencia, Spain; ^4^Head of Rheumatology Division, Hospital Universitario Central de Asturias, Oviedo, Spain

**Keywords:** axial spondyloarthritis, NSAIDs, therapeutic goals, gut dysbiosis, long-term prognosis, disease activity, disease impact

## Abstract

Non-steroidal anti-inflammatory drugs (NSAIDs) remain the mainstay of treatment for spondyloarthritides (SpA), a group of entities with common clinical and pathophysiological aspects, but also with differential features. Although NSAIDs provide significant symptomatic relief, especially for joint pain and morning stiffness, their role in achieving and maintaining the treatment goals advocated by the treat to target strategy in SpA is not entirely clear. These agents can induce changes in the composition of the intestinal microbiota, also favoring an alteration of the barrier function in the gut epithelium. All of this, favored by a pre-disposing genetic background, could activate a specific type of aberrant immune response in the gut lamina propria, also known as type-3 immunity. This article offers a perspective on how NSAIDs, despite their undeniable value in the short-term SpA treatment, could hinder the achievement of medium and long-term treatment goals by compromising the barrier function of the gut mucosa and potentially altering the composition of the gut microbiota.

## Introduction

Spondyloarthritides (SpA) comprise a group of entities with common features, but also with differential facts. The Assessment of SpondyloArthritis International Society (ASAS) group has provided a unifying conceptual framework dividing these entities based on the dominant clinical picture into axial and peripheral forms. Within the former, we find radiographic and non-radiographic forms of axial SpA (axSpA), and among the latter, psoriatic arthritis, and others. Spondyloarthritides affect a variable proportion of subjects within the general population, and its prevalence is clearly associated with the presence of HLA-B27 in the target population. Furthermore, these diseases seriously affect the quality of life and social participation, so the impact they generate on patients and health care systems is enormous. Although non-steroidal anti-inflammatory drugs (NSAIDs) continue to be the mainstay of treatment for these conditions, in recent years different biological drugs, as well as Janus kinase inhibitors, have entered the SpA therapeutic market. This growing complexity is leading to questioning the current way of approaching the disease from a pharmacological point of view (1).

## NSAIDs: Lights and Shadows

NSAIDs are widely used by the general population due to their high efficacy for treating pain, inflammation, and fever, both in children and adults (2). They currently account for 5% of all drug prescriptions globally (2). Furthermore, we are currently seeing how the canonical uses of NSAIDs are giving way to emerging applications as potential antineoplastic, antiparasitic, antibacterial, and antidiabetic drugs (2). In the field of rheumatic diseases, they have been shown to be especially effective in treating pain, joint stiffness, inflammation, and functional limitation, associated with inflammatory conditions such as early arthritis, gout, or axSpA (3–5). In these conditions, we know of the successful use of NSAIDs since the 1950s (6). The efficacy of NSAIDs in patients with axSpA is such that they are the first drugs recommended for the management of this entity (5), and in fact, the rapid response to NSAIDs is considered one of the minor criteria within the classification framework for these diseases proposed by the ASAS group (7).

High to moderate-quality evidence indicates that both traditional and cyclooxygenase-2 specific inhibitor NSAIDs are efficacious for treating axSpA, and moderate to low quality evidence indicates harms may not differ from placebo in the short term (8). Furthermore, different NSAIDs seem equally effective in this regard (8). On the other hand, continuous rather than on-demand NSAIDs use may be effective in retarding radiographic progression in axSpA, especially in patients with high disease activity and higher C-reactive protein levels (9).

Although the ASAS-European League Against Rheumatism (EULAR) recommendations advocate continued vs. on-demand use in patients with active SpA (5), the long-term use of NSAIDs is limited by their potential deleterious effects. Thus, NSAIDs are associated with 30% of hospital admissions for preventable adverse drug reactions (2). Therefore, despite the undeniable success of NSAIDs, the price to pay for such success can be very onerous in the middle and long term. It is known that NSAIDs are associated with an increased risk of cardiovascular toxicity among general and arthritic population. However, the reduction of systemic inflammation in inflammatory arthritis may reduce cardiovascular morbidity and mortality. In that line, a lower cardiovascular event rate in NSAID users has been reported in axSpA (10).

## NSAIDs: Gut Dysbiosis and Type-3 Immunity

The chronic use of NSAIDs has been associated with different major organ damage, one of the main ones being damage to the gastric mucosa which also may extend to the lower gastrointestinal tract (2, 11). Upper and lower digestive tract mucosal damage is in part due to the inhibition of prostaglandin synthesis exerted by NSAIDs (2, 11). However, other mechanisms of damage have been invocated. Thus, NSAIDs may have some cytotoxic effects through a mitochondrial oxidative stress-based mechanism, a pathological process characterized by a severe mitochondrial damage due to the activation of detrimental redox-active chain reactions which is accompanied by impairment to the cellular energy generation mechanisms and eventual cell death (2, 11). Uncoupling of mitochondrial oxidative phosphorylation, adenosine triphosphate (ATP) deficiency, elevation of cytosolic Ca2+ and Na+/K+ imbalance and consequent induction of apoptosis are some of the hallmark and common events triggered in both these gut compartments by NSAIDs. One of the final consequences of this process is a potentially severe alteration of the barrier function of the intestinal epithelium (2, 11). Interestingly, NSAIDs use can affect the gut microbiota composition and metabolic activity through a direct effect on the microbiota (by inhibiting/facilitating microbial growth, inducing microbial cell death and/or influencing microbial metabolism) or through an indirect effect by interacting with the host (by changing the metabolism, gut environment, mucosal integrity, and permeability) which may, in turn, precipitate in dysbiosis (12). For example, treatment with aspirin causes a shift in the composition of the gut microbiota regarding *Prevotella, Bacteroides, Ruminococcaceae*, and *Barnesiella*, whereas celecoxib and ibuprofen increase the abundance of *Acidaminococcaceae* and *Enterobacteriaceae*. Ibuprofen causes enrichment in *Propionibacteriaceae, Pseudomonadaceae, Puniceicoccaceae*, and *Rikenellaceae* species compared with either non-users or naproxen users (12). Therefore, NSAID use induces mainly the overgrowth of Gram-negative and anaerobic bacterial species, which, possibly through the release of endotoxin or microbial metabolites, lower mucosal defense and increase the susceptibility to intestinal damage. Also, increased intestinal permeability, migration of bacteria through the epithelium into the deeper layers of the mucosa, and mucosal inflammatory and immune response can be observed when the mucosal barrier function is disrupted by NSAID-mediated topical effects and prostanoid inhibition (11, 12). Moreover, lipopolysaccharide (LPS) and high mobility group box 1 (HMGB1), when present in the lumen, can activate NLRP3 inflammasome through the binding to Toll-like receptor 4 (TLR4) in the intestinal cells, causing inflltration of neutrophils and macrophages and resulting in deeper ulceration of the small intestinal mucosa, which in turn supposes a positive feedback mechanism for further damage to the intestinal mucosa (11, 12). In any case, changes in the composition of the gut microbiota may be influenced by other factors such as the concomitant use of other drugs (for example, proton pump inhibitors can even potentiate the damage to the gut mucosa generated by NSAIDs), age, or sex of patients under treatment with these drugs (12).

**Figure 1:**
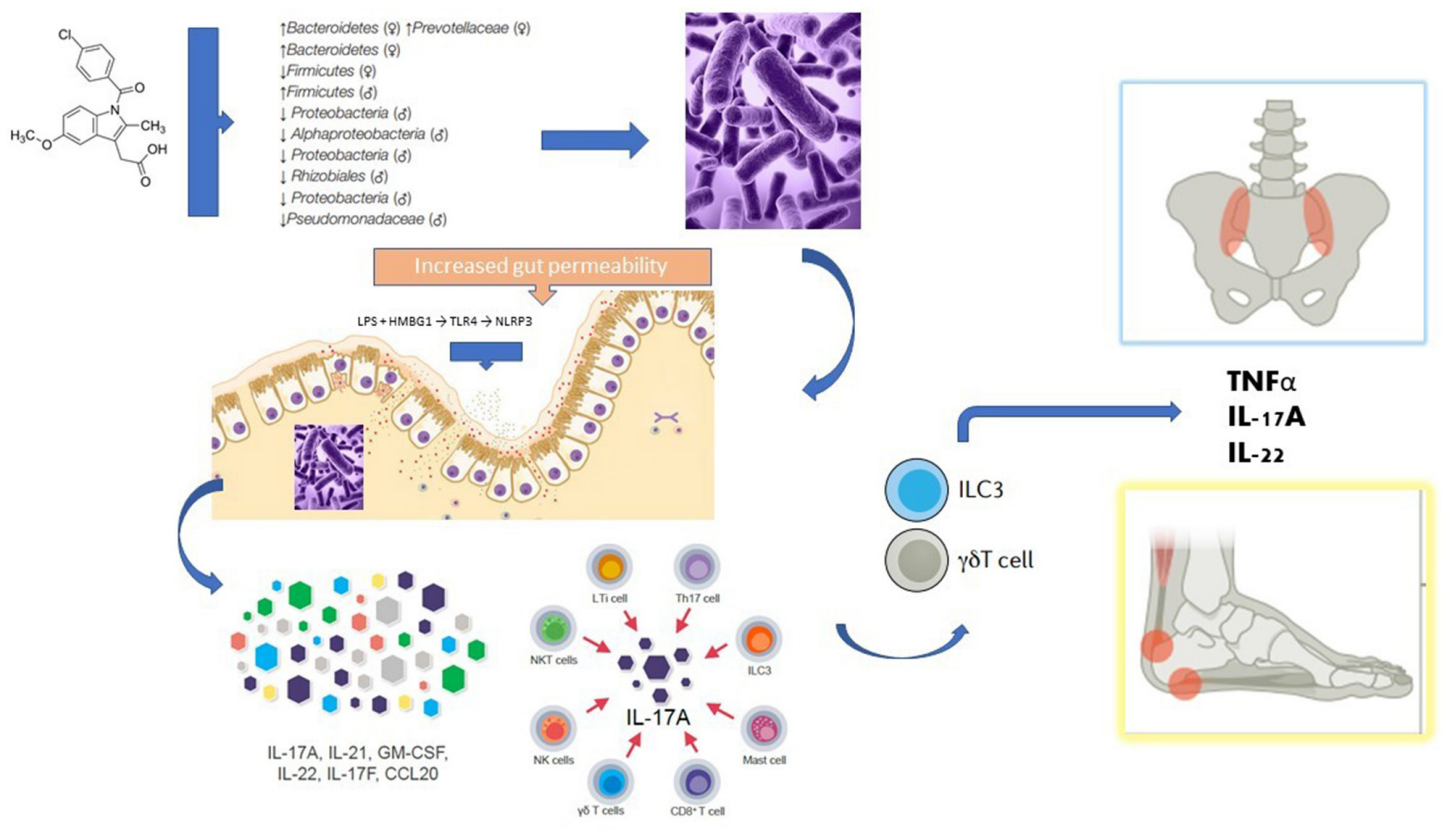
Potential mechanisms linking the use of NSAIDs with the gut-joint axis theory in the pathogenesis of spondyloarthritis. NSAIDs (in this case exemplified by indomethacin) can modify the growth and imbalance the composition of the intestinal microbial communities (a condition known as dysbiosis). Once substantial damage is generated to the defense mechanisms of the intestinal mucosa, and pathogenic bacteria and their products reach deeper layers of the intestine, it is believed that a special type of immune response called type 3 immunity is activated in the lamina propria. In SpA patients, several cell lineages with the potential to produce IL-17 are increased in the blood, including mucosal-associated invariant T cells, Th17 cells, γδT cells and type 3 innate lymphoid cells (ILC3), all of which have been implicated in mucosal immunity. Human entheseal tissue contains IL-23-responsive γδT cells and ILC3 cells and these cells are important producers of IL-17 and IL-22 in the blood and peripheral joint synovial fluid of patients with SpA. It is believed that these type 3 cells can reach distant joint structures through the bloodstream, whereby secreting proinflammatory cytokines they can generate synovitis, osteitis, and enthesitis (gut-joint axis). See text for a more detailed explanation.

Changes in gut microbiota composition are correlated with autoimmune diseases through the activation of the immune response, molecular mimicry, and increased intestinal permeability, among other mechanisms (13). Spondyloarthritis patients have a distinct gut microbiota compared to healthy controls, and 60–70% of patients with SpA present microscopic evidence of gut inflammation (13). Dysbalances in *Lachnospiraceae, Veillonellaceae, Prevotellaceae, Porphyromonadaceae*, and *Bacteroides* spp. have been reported in patients with axSpA (13). However, these microbiome alterations seem strongly influenced by the genetic background. In that sense, different gut microbiome compositions were detected in healthy HLA-B^*^27 positive and negative individuals, which supports the importance of host genetic background in shaping the gut microbiome (13). Either by mostly genetic factors or by factors more dependent on the host or its environment, it is conceivable that gut dysbiosis, and the subsequent increased intestinal permeability, may enhance translocation of microbial products to the lamina propria favoring a type 3 immunity response (also referred to as “type Th17 immunity” or “IL-17-associated immunity”) that subsequently migrates to peripheral tissues, including uvea, joints, and entheses (14). In fact, human entheseal tissue contains IL-23-responsive γδT cells and ILC3 cells (type-3 cells) and these cells are important producers of IL-17 and IL-22 in the blood and peripheral joint synovial fluid of patients with SpA (14). The finding of α4β7 integrin overexpression, an intestinal homing factor, in the synovial tissue of patients with SpA favors this hypothesis (14). This causal hypothesis of the gut–joint axis of inflammation provides a theoretical explanation for how perturbed immunity and microorganisms interact to initiate a type 3 inflammatory cascade that can spread to the joint and other extraintestinal sites (Figure 1). However, dysbiosis and associated intestinal inflammation are not universal findings in patients with SpA. It is possible, therefore, that the inflammation observed in the joints and gut of patients with SpA, are only two phenomena that share immunoinflammatory mechanisms (type 3 immunity), but not necessarily a causal link (14).

## NSAIDs and Treatment Goals: Moving Closer or Further Away?

NSAIDs remain the mainstay of axSpA treatment. However, chronic NSAIDs use could potentially favor a state of gut dysbiosis, which in turn, would lead to positive feedback on type 3 immune responses which are at the basis of the gut-joint axis theory, as discussed earlier. The frequent exacerbation of inflammatory bowel disease after continued use of NSAIDs may be a clinical endorsement of this hypothesis (15). Obviously, clinical experience teaches us that chronic use of NSAIDs is not linked to a higher frequency of overt inflammation at joints or entheses in patients with axSpA, but the question herein is whether patients with long-standing axSpA who receive NSAIDs on a regular basis are associated with a higher or lesser probability of achieving therapeutic goals or of being in a state of higher or lower disease impact. Recent observational studies, both cross-sectional and longitudinal, have explored these relationships. As reflected in the Groningen Leeuwarden Ankylosing Spondylitis (GLAS) cohort, after a 52-week follow-up period, higher NSAID intake was related to higher Ankylosing Spondylitis Disease Activity Score (ASDAS) and vice versa, regardless of use of TNF-alpha inhibitors (16). Moreover, the regular use of NSAIDs has been associated with a 5-fold increase in the odds of being in a higher disease impact category according to the ASAS health index, once again independently of biological drug use (17). Finally, in a recent study, after controlling for several confounders (including biologics use), NSAIDs intake was independently related to the odds of not reaching therapeutic goals as expressed by the Bath Ankylosing Spondylitis Disease Activity (BASDAI) remission criteria (OR 0.18), ASDAS inactive disease (OR 0.08), and Routine Assessment of Patient Index Data 3 (RAPID3) remission (OR 0.26) (18). Anyway, these observational studies are potentially exposed to several biases that may lead to misinterpretation of their results. One of the main biases in such studies is due to confounding by severity, given that axSpA patients who receive NSAIDs on a regular basis are likely those more severely affected by the disease; despite this, biological therapy should have been subject to similar bias in these studies, but this was not the case.

## Discussion

The ASAS-EULAR management recommendations for axSpA advocate for adequate control of symptoms and signs of disease, delaying the progression of structural damage, but at the same time maximizing the quality of life as well as the social participation, limiting or avoiding the risks associated with the use of the drugs indicated for these entities (5). NSAIDs can certainly help control the signs and symptoms of active SpA and potentially delay axial radiographic progression. However, their role in achieving the treatment goals of remission or low ASDAS disease activity included under the treat to target (T2T) strategy proposed by EULAR for axial and peripheral SpA (19) seems less clear. Furthermore, its role in optimizing the health-related quality of life of these patients is also in question, as we mentioned before.

Currently, we are witnessing a change in the management paradigm for axSpA. Thus, the first studies adopting a T2T strategy for axSpA are already underway (20). The preliminary results of these studies suggest that treatment with the increasingly early introduction of biological therapy could better fit the EULAR management recommendations for axSpA (20). Apart from their short-term symptom control role, we need to rethink where we place NSAIDs in the medium and long-term disease management. Until this is achieved, it is imperative to carefully weigh the advantages and disadvantages of NSAIDs before considering their long-term use (Table 1).

**Table 1:** Potential advantages and disadvantages of NSAIDs for treating axial SpA.

**Advantages**	**Disadvantages**
Highly effective in relieving joint pain, inflammation, and joint stiffness.	Potential for major organ damage (upper/lower gastrointestinal tract, liver, cardiovascular, kidney, etc.)
Useful to improve physical function allowing patients to be able to carry out other therapeutic measures (e.g., physiotherapy)	Alteration of intestinal permeability favoring dysbiosis
Potential to delay axial radiographic damage (on continued use)	Potential for positive feedback on a gut-joint axis mediated by type-3 immunity response
Cardiovascular benefits of reducing systemic inflammation may outweigh the overall cardiovascular toxicity	Negative association with the achievement of treatment goals in observational studies
Emerging applications^*^	Negative association with a low disease impact in observational studies

* *Antineoplastic, antiparasitic, antibacterial, and antidiabetic*.

## Data Availability Statement

The original contributions presented in the study are included in the article/supplementary material, further inquiries can be directed to the corresponding author.

## Author Contributions

Material preparation, literature review, and analysis were performed by RQ-S, AG-V, SA-C, and MA-L. The first draft of the manuscript was written by RQ-S. All authors contributed to the study conception and design, commented on previous versions of the manuscript, and read and approved the final manuscript.

## Conflict of Interest

The authors declare that the research was conducted in the absence of any commercial or financial relationships that could be construed as a potential conflict of interest.

## Publisher's Note

All claims expressed in this article are solely those of the authors and do not necessarily represent those of their affiliated organizations, or those of the publisher, the editors and the reviewers. Any product that may be evaluated in this article, or claim that may be made by its manufacturer, is not guaranteed or endorsed by the publisher.
